# Quantum Anomalous Hall Effect in Graphene-based Heterostructure

**DOI:** 10.1038/srep10629

**Published:** 2015-05-29

**Authors:** Jiayong Zhang, Bao Zhao, Yugui Yao, Zhongqin Yang

**Affiliations:** 1 State Key Laboratory of Surface Physics and Key Laboratory for Computational Physical Sciences (MOE) & Department of Physics, Fudan University, Shanghai 200433, China; 2 School of Physics, Beijing Institute of Technology, Beijing 100081, China; 3 Collaborative Innovation Center of Advanced Microstructures, Fudan University, Shanghai, 200433, China

## Abstract

Quantum anomalous Hall (QAH) effect, with potential applications in low-power-consumption electronics, is predicted in the heterostructure of graphene on the (001) surface of a real antiferromagnetic insulator RbMnCl_3_, based on density-functional theory and Wannier function methods. Due to the interactions from the substrate, a much large exchange field (about 280 meV) and an enhanced Rashba spin-orbit coupling are induced in graphene, leading to a topologically nontrivial QAH gap opened in the system. The avenues of enhancing the nontrivial gap are also proposed, from which nearly a gap one order large is achieved. Our work demonstrates that this graphene-based heterostructure is an appropriate candidate to be employed to experimentally observe the QAH effect and explore the promising applications.

Finding topologically nontrivial states in realistic materials has attracted considerable attention recently in condensed matter physics and materials science^1^. Ferromagnetic (FM) order, spin-orbit coupling (SOC), and special band structures can generate quantum anomalous Hall (QAH) effect^2^, showing promising applications in low-power-consumption electronic devices. The experimental observations of QAH effects have been reported in topological insulator films of (Bi,Sb)_2_Te_3_ with Cr magnetic atoms doped^3^,^4^,^5^. The observation temperature, however, is very low (milli-Kelvin) probably associated with the relatively weak long-range FM order and disorder effects from the magnetic dopants etc. Some theoretical proposals were also raised to realize the QAH effect^6^,^7^,^8^,^9^,^10^,^11^,^12^,^13^,^14^,^15^,^16^,^17^,^18^ in different systems, including Hg_1-y_Mn_y_Te^6^, graphene^7^,^8^,^9^,^10^, silicene^11^,^12^,^13^, MnTe magnetic substrates deposited with Pb atoms^15^, organic TIs^16^, and half-hydrogenated Bi honeycomb monolayers^17^. Both the experimental and theoretical researches indicate that forming a long-range FM order is one of the most important and tough tasks in carrying out the QAH effect.

Nowadays, graphene has become a prototype material for engineering the QAH effect^7^,^8^,^9^,^10^ because of its unique homeycomb lattice, special electronic properties, and the relatively mature technologies of sample growth and device fabrication. Pristine graphene is a topological insulator^19^ but with a very small nontrivial band gap due to its extremely weak intrinsic SOC^20^,^21^. The time-reversal symmetry of graphene can be broken by introducing an external magnetic exchange field through adsorbing transition metal (TM) atoms^7^,^8^,^9^,^10^. The Rashba SOC strength in graphene^7^ can also be enhanced effectively through the hybridization of graphene C *p*_z_ and TM *d* states^9^,^10^,^22^,^23^,^24^. The adsorption energies of TM atoms on graphene are, however, usually very small^9^ and the TM atoms tend to form into clusters^25^,^26^. Thus, the long-range FM order may not survive as expected. Finding new strategies to generate the QAH effect in graphene and related honeycomb-lattice systems are of significance^23^,^27^,^28^.

In this work, we tend to achieve the QAH state in the heterostructure of graphene on the (001) surface of a real antiferromagnetic (AFM) insulator RbMnCl_3_, which has an AFM structure with the Mn sites forming alternating (001) FM planes. Through the proximity effect, the topmost FM Mn plane in RbMnCl_3_ may induce a strong FM exchange field in graphene, a key factor to achieve the QAH effect. This material can be grown easily by molecular beam epitaxy methods in experiments, whose (001) surface also has a hexagonal structure^29^,^30^ as the graphene does. Besides, its lattice can match well with the graphene lattice. Therefore, RbMnCl_3_ could be a suitable substrate for graphene to gain the QAH state. Based on density-functional theory (DFT) and Wannier function methods, we demonstrate the QAH effect in graphene and (001) RbMnCl_3_ heterostructure. A much larger exchange field (about 280 meV) is obtained in graphene. Berry curvature, Chern number, and anomalous Hall conductivity are calculated to confirm the topologically nontrivial band gap opened by the SOC. Our results demonstrate that this heterostructure is an appropriate candidate system for exploring the QAH effect and corresponding electronic applications.

The RbMnCl_3_ compound has a hexagonal structure with the experimental lattice constant of a = 7.16 Å^30^, which is adopted in our calculations. A 3 × 3 supercell of graphene was employed to match the (001) surface of RbMnCl_3_ with Mn terminal, as illustrated in Fig. 1(a). The mismatch between the graphene and RbMnCl_3_ lattices is about 2.5%. In our calculations, the graphene lattice is shrunk to fit the substrate lattice. The C atoms in graphene and the topmost Mn, Rb, and Cl atoms in the substrate were all relaxed to obtain the stable structure of the heterostructure. The magnetization of the Mn atoms close to the graphene in the heterostructure is found to be along the *c*-axis. Its energy is lower than that of the magnetization in the *ab* plane by 0.25 meV per unit cell. Three possible adsorption configurations were considered: the hollow, bridge, and top sites, which stand for the topmost Mn atoms of the substrate right below the hexagon centers (hollow sites), the hexagon edges (bridge sites), and the hexagon corners (top sites) of the graphene, respectively. The relative stability of the three configurations is judged by comparing their adsorption energies, defined as E_b_ = E_G_ + E_R_ − E_G+R_, where E_G_, E_R_, and E_G+R_ represent the total energies of the bare graphene supercell, the bare RbMnCl_3_ film, and the graphene/RbMnCl_3_ heterostructure, respectively. The hollow site is found to be the most stable configuration, displayed in Fig. 1(a) and Fig. 1(b) and employed in our following discussion. The calculated adsorption energy of 0.99 eV for the hollow site indicates a relative strong interaction between graphene and the substrate. This tendency is consistent with the not very large average distance d_0_ (d_0_ = 2.03 Å) between the graphene plane and the topmost layer of Mn atoms in the substrate after the geometry optimization.

Figure 1(c) shows the reciprocal momentum space structures for the 1 × 1 and 3 × 3 supercells of graphene. The K and K’ points in the 1 × 1 cells are both folded into the Γ point of the 3 × 3 supercells. When the graphene adsorbed on the (001) surface of RbMnCl_3_ substrate, it is magnetized by the proximity coupled effect with the topmost ferromagnetic Mn layer. Figure 2(a) plots the band structure of graphene on the (001) surface of RbMnCl_3_ film. We find that the Dirac bands of graphene are just located inside the bulk band gap of the RbMnCl_3_ film. Obvious spin splitting appears in the bands around the Dirac point. Since the K and K’ Dirac points of the pristine graphene are both folded into the Γ point of the 3 × 3 supercell of graphene, each spin-up and spin-down Dirac bands around the Γ point are twofold degeneracy. Magnifying the spin-polarized π bands around the Dirac point at the Γ point (Fig. 2(b)), we can observe a very distinct exchange field (about 280 meV) generated in graphene by the nearest neighboring FM Mn layers of the substrate. Due to this proximity-induced exchange splitting, the spin-up and spin-down bands of the π states of the graphene cross each other around the Dirac point, which is essential to result in the QAH effect^12^,^14^. The corresponding band structure with SOC is shown in Fig. 2(c) and Fig. 2(d). A bulk band gap of about 1.2 meV is opened at the crossing points of the spin-up and spin-down Dirac bands, seen explicitly from the magnified band in Fig. 2(d). This band gap is much larger than the topologically nontrivial gap of pure graphene (24 × 10^−3^  meV)^20^ due to the enhanced Rashba effect in the π bands of graphene through the hybridization with the Mn 3*d* states in the substrate^22^. If the on-site Coulomb interaction U is neglected, the contribution of the Mn *d* orbit to the Dirac bands increases, leading to a larger exchange field and Rashba SOC. Thus, the opened bulk band gap could increase slightly to about 2.0 meV in this case.

The mechanism of the gap opening can be understood by a tight-binding (TB) model of graphene with a TM adsorbed at the hollow site in a 3 × 3 supercell. We consider the TM-induced on-site potential, magnetic exchange field, and Rashba SOC on the six carbon atoms nearest to the TM adatoms. The TB Hamiltonian can be written as^7^,^19^


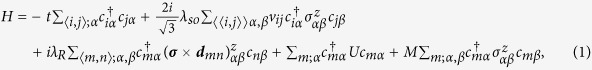


where 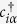 (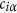) creates (annihilates) an electron with spin 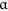 on site *i*, 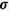 are the Pauli matrices, 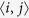
(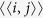) runs over all the nearest- (next-nearest)-neighboring hopping sites, 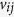 = 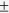
1 if the nest-nearest-neighboring hopping is counterclockwise (clockwise) with respect to the positive *z* axis, *m*(*n*) refers to the sites nearest to the TM atom, and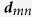 denotes lattice vector pointing from site *m* to site *n*. The first term represents the nearest-neighboring hopping with amplitude *t* = 2.6 eV, the second term is the intrinsic spin-orbit coupling. The last three terms correspond to the TM-induced extrinsic Rashba SOC, on-site potential and magnetic exchange field, respectively. For the TM-induced intrinsic SOC is usually much smaller than the induced Rashba SOC, we neglect the intrinsic SOC term by setting 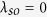. Figure S1(a) plots the band structure of pristine 3 × 3-graphene with the perfect Dirac bands folded into the Γ point. We can observe a trivial band gap opened at the Γ point in the presence of only the on-site potential U for the coupling of valleys K and K’ (while no trivial band gap opens in the 4 × 4 supercell of graphene in this case) as shown in Fig. S1(b). When a large exchange field M is included, the spin-up and spin-down Dirac bands around Γ point cross with each other and the gap disappears, as displayed in Fig. S1(c). A new band gap can be opened at the band-crossing points after Rashba SOC 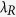 considered, explaining well the band gap obtained from the first-principles calculations (Fig. 2(d)).

The Berry curvature and Chern number are calculated to identity whether the SOC-induced gap is topologically nontrivial. The Berry curvature Ω(**k**) is calculated by^31^,^32^,^33^,^34^,^35^,^36^:


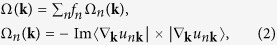


where the summation is over all the *n* occupied bands,
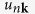
is the periodic part of the Bloch wave in the *n*th band, 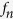 is the Fermi-Dirac distribution function. In this work, the Berry curvature is calculated on the basis of the Wannier functions. We first constructed the maximally localized Wannier functions (MLWF)^37^,^38^ via a non-self-consistent calculations on 8 × 8 × 1 k-point grids based on the previously converged DFT self-consistent charge potential, as implemented in the Wannier90 package^39^. We then adopted the numerical algorithm in Ref. [40] to calculate the Berry curvature Ω(**k**). For two-dimensional systems, the calculated Berry curvature is the z-component value. The Chern number C and Hall conductivity σ_xy_ can be obtained by integrating the Ω(**k**) over the first BZ, as 
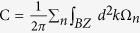
 and 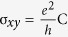. The red dotted curve in Fig. 3(a) shows the Berry curvature of the graphene/RbMnCl_3_ heterostructures. The peaks of the Berry curvature are primarily located at the k points with the SOC-induced band gaps. The corresponding distribution of Ω(**k**) in the two-dimensional (2D) momentum space is given in Fig. 3(b), indicating clearly that Ω(**k**) distributes very close to the Γ point in the first BZ. Integrating the obtained Ω(**k**) over the first BZ, the Chern number of C = 2 is obtained. This integer Chern number (C = 2) characterizes a quantized Hall conductivity and confirms the topological non-triviality of the gap opened by the SOC interaction. Two chiral edge channels will arise on each side of the sample. Therefore, the QAH effect can appear in the graphene/RbMnCl_3_ heterostructure. Since the nontrivial gap is opened below the Fermi energy (E_F_), hole doping is needed to observe this exotic effect in experiments. Our first-principles calculations indicate that doping one hole per unit cell could move the E_F_ to the crossing points of spin-up and spin-down bands, as shown in Fig. 3(c). The corresponding hole doping concentration is about 2.2 × 10^14^ cm^−2^, which can be experimentally achieved through currently advanced gating technologies. For instance, employing an electrolytic gate, the doping concentration in graphene can be adjusted up to 4 × 10^14^ cm^−2^ in experiments^41^. When the SOC is further considered, the topologically nontrivial bulk band gap can be opened just around the E_F_.

If the interaction between the graphene and RbMnCl_3_ substrate is strengthened, the exchange field and Rashba SOC in the graphene can be enhanced. To increase the interaction, we reduce the distance between the graphene sheet and the substrate, by applying an externally vertical stress to the heterostructure. As shown in Fig. 3(d), the SOC-induced topologically nontrivial band gap is indeed enlarged due to the strengthening of the exchange field and Rashba SOC interaction in the graphene. The gap increases obviously with the increase of the vertical stress. When the distance is reduced by 10%, the band gap E_g_ can be almost doubled (2.1 meV). In experiments, the exerted stress, however, cannot be too large. The structure of the systems may be destroyed under a very large strain.

Other than exerting vertical stress, doping heavy atoms to the graphene is also explored to enlarge the Rashba SOC interaction in the heterostructure. Since the FM order of graphene sheet in the studied system has already been triggered, only heavy atoms are needed. We take 5d TM Hf atom as an example to investigate the effect. The calculated most stable adsorption site is the hollow site of graphene above the Rb atom, as shown in Fig. 4(a), with the adsorption energy larger than 1.7 eV. Figure 4(b) shows the band structure of the graphene/RbMnCl_3_ heterostructure with Hf atoms adsorbed at the hollow site of the graphene. A relatively large band gap of about 9.0 meV appears in the system. Thus, compared to the original gap, nearly one order large gap is achieved by adding Hf atoms into the system. The origin of this large band gap comes from the enhanced Rashba SOC due to the adsorption of Hf atoms, as reported in Refs. [9,^22^]. To check the possible electric-field effect induced by the adsorbed Hf atoms, a large externally vertical electric field of 0.3 V/Å is applied to the system. The obtained nontrivial gap nearly remains the same as the value without the electric field. Thus, the effect of the electric field is negligible for the gap opening in the adsorption system. The complete band structures without and with SOC are shown in Fig. S2. The maximally localized Wannier functions of this adsorbed system are also constructed. The calculated Berry curvature Ω(**k**) and its corresponding 2D distribution in momentum space are illustrated in Fig. 4(b) and Fig. 4(c), respectively, where the E_F_ is adjusted in the gap. The Ω(**k**) peaks are also localized around the SOC-induced band gap near the Γ point. The intrinsic anomalous Hall conductivity σ_xy_ as a function of the E_F_ is plotted in Fig. 4(d). The quantized Hall conductivity platform appears when the E_F_ passes the SOC-induced bulk band gap, confirming the existence of the QAH effect in this proposed system. The Hall conductivity in Fig. 4(d) changes sign when E-E_F_ is larger than −1.24 eV, ascribing to the spin-direction reversal of the main bands around this energy point (see Fig. S2(c)).

To investigate the case of a lower adsorption concentration, which may be closer to the experimental situation, we adopted a large 6 × 6 supercell of graphene on a 2 × 2 supercell of RbMnCl_3_ with one Hf atom adsorbed. The calculated band structures were plotted in Fig. S3. It is convinced that the QAH effect still exist in low adsorption concentration. Thus, it is desirable to observe experimentally the QAH effect with large gaps in the system with low adsorption concentrations to avoid the interactions between the adatoms. The above proposed topologically nontrivial bulk band gap corresponds to a temperature around 100 K, which is promising for high temperature operations and practical applications of graphene-based QAH effect. Finding suitable substrate materials with heavier elements may lead to a larger Rashba SOC in the graphene. Hence, a larger nontrivial band gap may be achieved in the heterostructure.

In summary, we systematically investigated the QAH effect of graphene deposited on the (001) surface of an AFM insulator RbMnCl_3_ from first-principles calculations. The calculated Berry curvature, Chern number, and anomalous Hall conductivity indicate that the system can present QAH effect. Two chiral edge channels will appear on each side of the sample. The topologically nontrivial QAH state can be detected in experiments through measurements of the quantized Hall conductivity platform or the edge states by injecting holes into the system. The nontrivial band gap can be increased significantly by reducing the distance between graphene and the RbMnCl_3_ substrate or adsorbing Hf atoms above the graphene plane. Our findings may push the experimental observations and practical applications of graphene-based QAH effect.

## Methods

**Figure 1:**
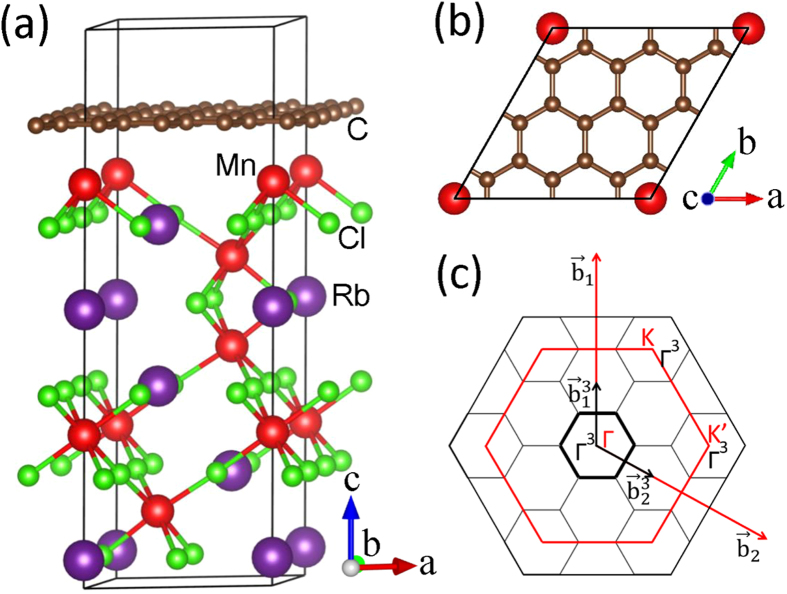
(a) Side view of the heterostructure with graphene on a (001) RbMnCl_3_ surface. The C, Rb, Mn, and Cl atoms are denoted in brown, purple, red, and green, respectively. (b) The top view of the heterostructure with the topmost layer Mn atoms of the MnRbCl_3_ located at the hollow site of graphene. For clarity, only graphene and the topmost layer Mn atoms are displayed. (c) The reciprocal momentum space structures for 1 × 1 and 3 × 3 supercells of graphene: 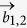 and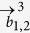 are for reciprocal vectors for 1 × 1 and 3 × 3 supercells, respectively. K and K’ points for the 3 × 3 supercell of graphene are folded into the Γ point.

The geometry optimization and electronic structure calculations were performed by using the first-principles method based on density-functional theory (DFT) with the projector-augmented-wave (PAW) formalism^42^, as implemented in the Vienna ab-initio simulation package (VASP)^43^. The Perdew-Burke-Ernzerhof generalized-gradient approximation was used to describe the exchange and correlation functional^44^. The substrate with different thicknesses containing three to six layers of Mn (Rb, Cl) planes are chosen (there are five Mn (Rb, Cl) layers in Fig. 1(a)). It is found that they give the same results. To stabilize the substrate, the Rb (Cl) terminal surface of the RbMnCl_3_ film, opposite to the graphene interface, was passivated by hydrogen atoms. A vacuum space of larger than 15 Å was used to avoid the interaction between two adjacent heterostructure slabs. For the structural relaxation, the C atoms in graphene and the topmost Mn, Rb, and Cl atoms in the substrate were allowed to relax until the Hellmann-Feynman force on each atom was smaller than 0.01 eV/Å. All calculations were carried out with a plane-wave cutoff energy of 550 eV and 12 × 12 × 1 Monkhorst-Pack grides were adopted for the first Brillouin zone (BZ) integral. To take into account the correlation effects of Mn 3d electrons, the GGA + U method^45^ was adopted and the value of the Hubbard U was chosen to be 3 eV. The van der Waals interaction (vdW) correction using the method of Grimme (DFT-D2)^46^ was considered in the calculations, including the structural relaxation.

## Additional Information

**How to cite this article**: Zhang, J. *et al.* Quantum Anomalous Hall Effect in Graphene-based Heterostructure. *Sci. Rep.* **5**, 10629; doi: 10.1038/srep10629 (2015).

## Supplementary Material

Supplementary Information

## Figures and Tables

**Figure 2:**
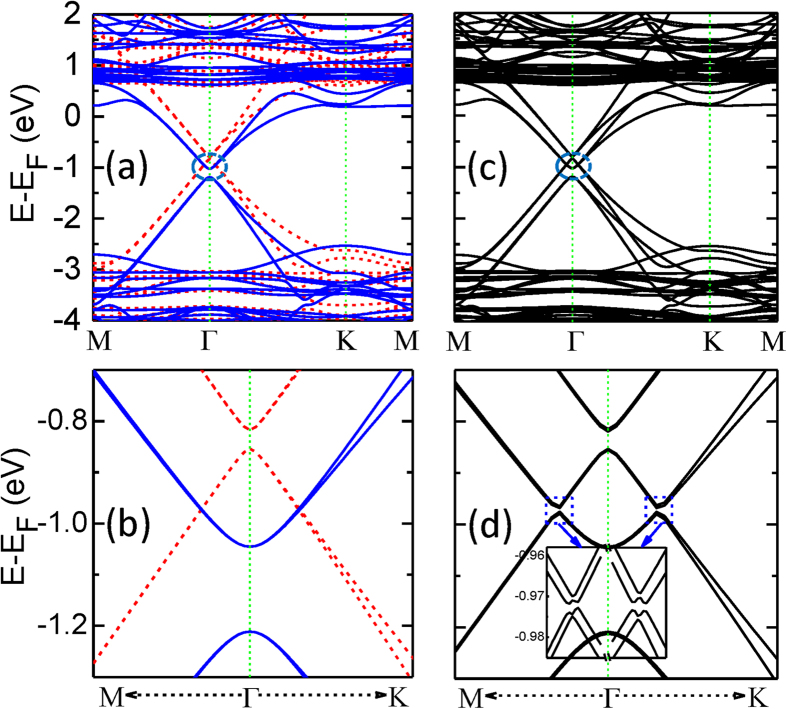
(a) and (c): Band structures of the heterostructure of 3 × 3 supercells of graphene on the RbMnCl_3_ substrate without and with the SOC interaction, respectively. Red dashed curves and blue solid curves denote the spin-up and spin-down bands, respectively. (b) and (d): Zoom in on the blue circles in (a) and (c), respectively. The inset in (d) is the magnified bands around the gap induced by the SOC interaction.

**Figure 3:**
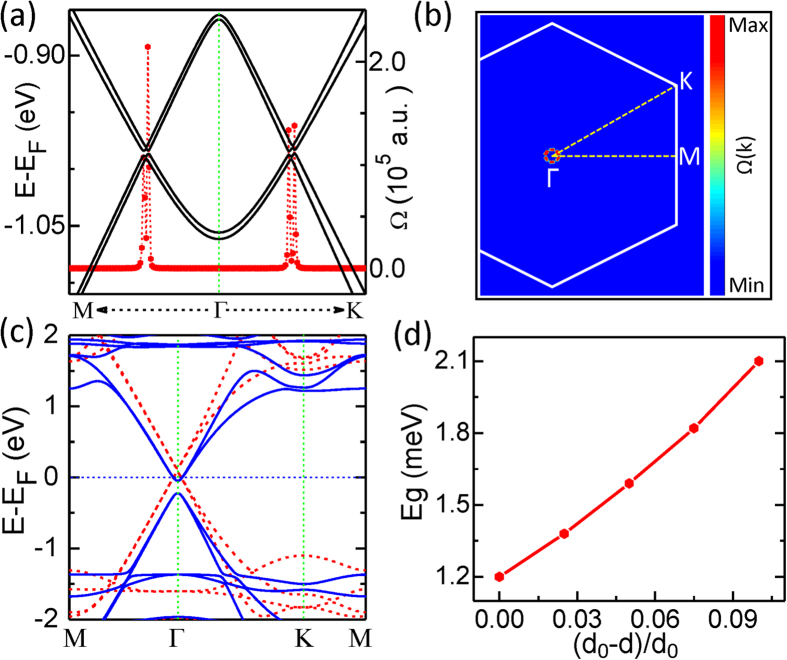
(a) The band structure (black solid curves) and the Berry curvature (red dotted curve) of the graphene/RbMnCl_3_ heterostructure. (b) The 2D distribution of the Berry curvature in the momentum space, located primarily around the Γ point. (c) The bulk band structure for the graphene/RbMnCl_3_ heterostructure with an extra hole in the supercell. (d) The bulk band gap Eg as a function of the change of (d_0_-d)/d_0_, where d is the distance between graphene and RbMnCl_3_ film and d_0_ is the corresponding optimized distance.

**Figure 4:**
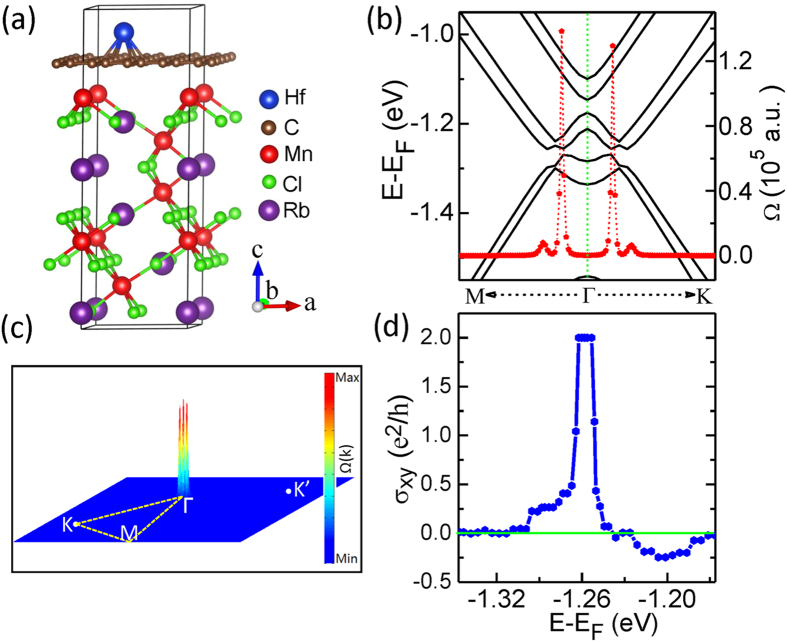
(a) Side view of the graphene/RbMnCl_3_ heterostructure with one adsorbed Hf atom per supercell, located at the hollow site of the graphene. (b) Band structures for the heterostructure displayed in (a) with SOC. The red dots denote Berry curvatures along high symmetry lines. (c) and (d): the distribution of the Berry curvature in the momentum space and the calculated anomalous Hall conductivity σ_xy_ as a function of the Fermi level for the graphene/RbMnCl_3_ heterostructure with Hf atoms adsorbed, respectively.
