# Supplementary material for: Quantum Anomalous Hall Effect in Graphene-based Heterostructure
Source: Sci Rep. 2015 May 29;5:10629. doi: 10.1038/srep10629 (PMC4448525; doi:10.1038/srep10629)
Supplement: Supplementary Information [file srep10629-s1.pdf]

Supplementary Information

Quantum Anomalous Hall Effect in Graphene-based Heterostructure

Jiayong Zhang[1], Bao Zhao[1], Yugui Yao[2], and Zhongqin Yang[1,3]*

[1]*State Key Laboratory of Surface Physics and Key Laboratory for Computational Physical Sciences (MOE) & Department of Physics, Fudan University, Shanghai 200433, China*

[2]*School of Physics, Beijing Institute of Technology, Beijing 100081, China*

[3]*Collaborative Innovation Center of Advanced Microstructures, Fudan University, Shanghai, 200433, China*

*Corresponding author: zyang@fudan.edu.cn

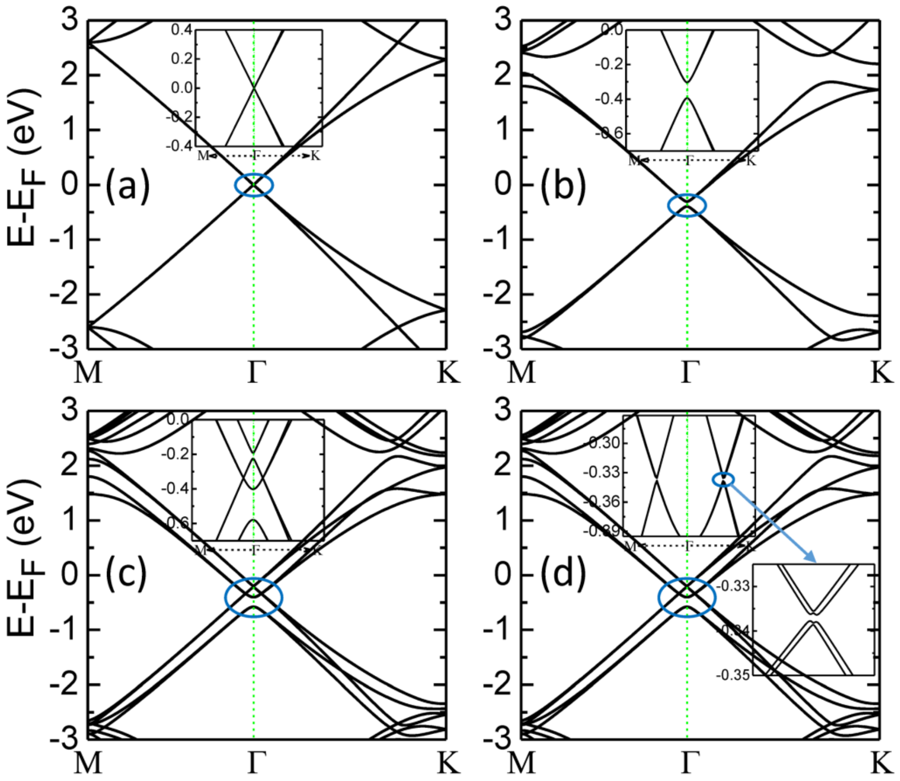

Figure S1. Band structures calculated by using TB model with graphene hopping strength t = 2.6 eV. The parameters adopted are (a) U= M =λ_R= 0. (b) U/t= -0.40, M =λ_R= 0. (c) U/t= -0.40, M/t = 0.16, λ_R= 0. (d) U/t = -0.40, M/t = 0.16, λ_R/t= 0.001. With these parameters, the bands around the Dirac points in (c) and (d) match well the Figure 2(a) and (c) obtained from the first-principles calculations, respectively.

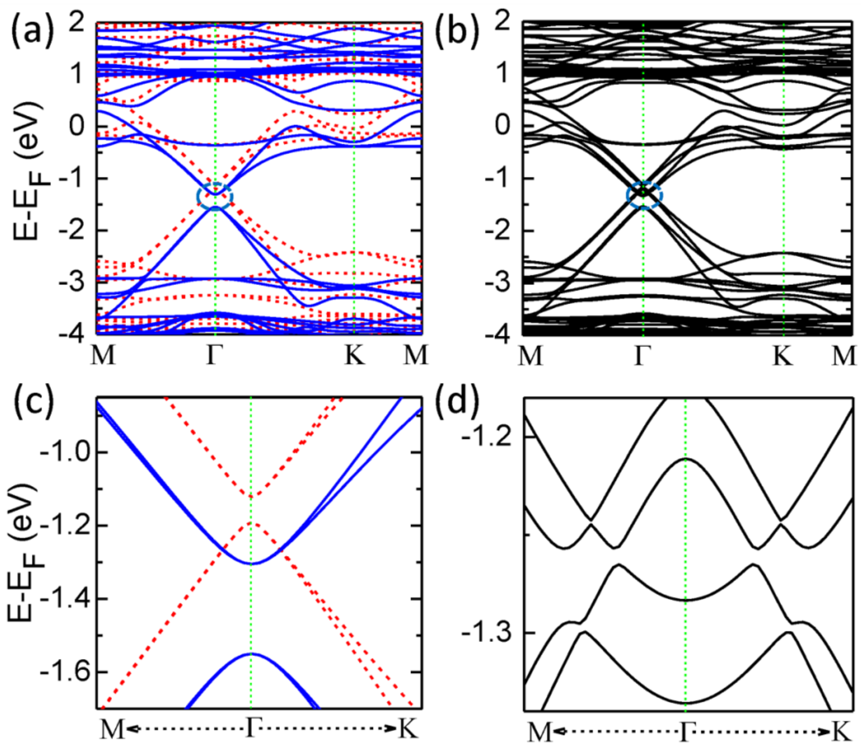

Figure S2. (a) and (b) The DFT band structures for the system with a 3×3 supercell of graphene on a 1×1 supercell of $MnRbCl_3$ without and with the consideration of the SOC interaction, respectively. One Hf atom is adsorbed in the supercell of the system, above the graphene plane. (c) and (d) Zoom in on the blue circles in (a) and (b), respectively.

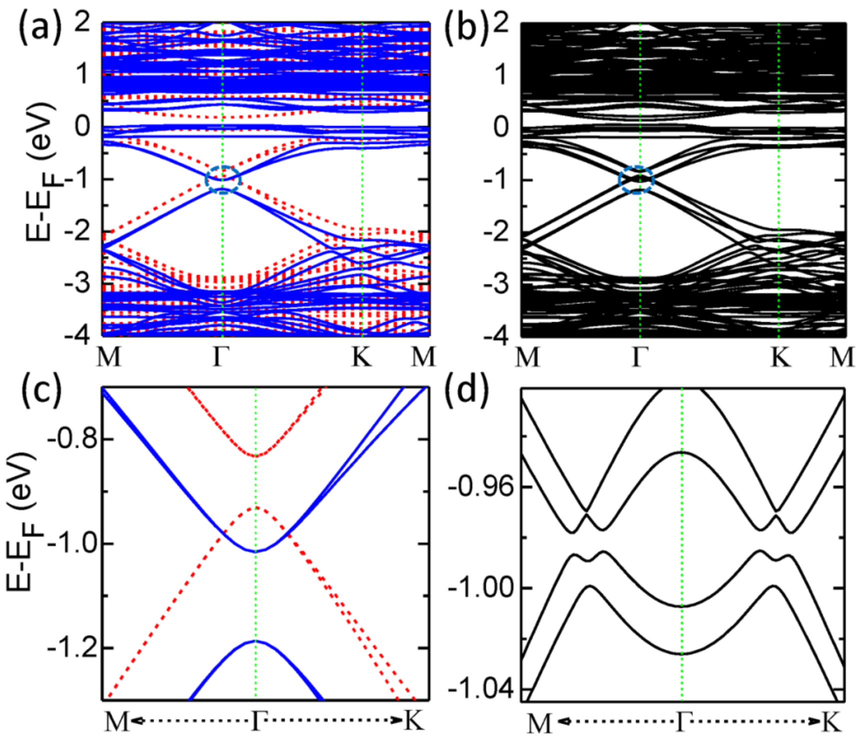

Figure S3. The same as **Figure S2.** except for the system with a 6×6 supercell of graphene on a 2×2 supercell of $MnRbCl_3$. One Hf atom is adsorbed in the supercell of the system, above the graphene plane.
